# A rare cause of acute liver failure- a case report

**DOI:** 10.1186/s12876-017-0730-6

**Published:** 2017-12-20

**Authors:** Sónia Bernardo, Sofia Carvalhana, Teresa Antunes, Paula Ferreira, Helena Cortez-Pinto, José Velosa

**Affiliations:** Departament of Gastroenterology and Hepatology, Hospital de Santa Maria, CHLN.Av. Prof. Egas Moniz, 1649-028 Lisbon, Portugal

**Keywords:** Acute liver failure, Diffuse malignant infiltration

## Abstract

**Background:**

Acute liver failure (ALF) induced by diffuse metastatic disease has rarely been reported.

**Case presentation:**

We present a 51-years-old woman with relevant clinical history for breast cancer. The patient was admitted in the emergency department with jaundice, dark urine and pale stools. She was on the 10th day of hormonotherapy for recurrence of breast cancer, diagnosed 7 years previously. Usual causes of acute liver failure were excluded, all drugs were stopped and the imaging studies performed were positive only for steatosis. Nonetheless, ALF progressed and the patient died 4 days later. Autopsy demonstrated a massive intrasinusoidal infiltration of the liver by breast cancer cells.

**Conclusion:**

We highlight a rare cause of ALF. Although uncommon, physicians should be alert for this situation as the diagnosis can be challenging and the imaging studies can remain normal.

## Background

ALF is defined by acute liver dysfunction manifesting as coagulopathy (INR ≥ 1.5) and presence of hepatic encephalopathy of any degree with less than 26 weeks duration, in a patient without preexisting liver disease [1].

The incidence of ALF is 2000 cases/year in USA [1]. The etiology varies broadly throughout the world and the most common causes of ALF are drug toxicity (50%), viral hepatitis (9%) and autoimmune hepatitis (7%) [2, 3]. However, in 20-40% of the cases the etiology remains unknown after an extensive workup [2].

The liver is a common target for metastasis, with 40% of autopsies in adults with malignant tumors, identifying liver metastases [4]. Nonetheless, a significant number of cases are asymptomatic with mild abnormal liver tests.

Malignancy is an uncommon cause of ALF and in very rare cases (0.44-1.4%) [3, 5] can occur due to a diffuse pattern of metastatic infiltration to the liver [3]. Different cancers have been associated with ALF [4]. Hematologic malignancies are the most common (41%), especially non-Hodgkin lymphoma [3, 4] but it also occurs with solid tumors, including breast (30%) [3], lung, pancreatic and gastric cancers for example.

The diagnosis of wide infiltration of the liver can be challenging because imaging studies are not able to detect this type of infiltration pattern and just 25% of the cases are diagnosed *premortem* [6]. Most cases have a poor prognostic, with death occurring in the majority and within several days of clinical presentation [2].

We report a case of ALF induced by metastatic infiltration of a breast cancer diagnosed 7 years before. We appraise the clinical and laboratorial findings, discuss treatment and prognosis, and review the available literature.

## Case presentation

We report a case of a 51 years-old woman with medical relevant story of ductal breast carcinoma, submitted to mastectomy followed by adjuvant radio and chemotherapy. She had remained disease-free for 7 years following treatment. After this period, due to tumor recurrence, the patient was started on hormonotherapy with Fulvestrant (estrogen receptor antagonist). Three days later, she developed asthenia, anorexia, nausea and vomiting. On day 10 of therapy she was admitted in the emergency department with jaundice, dark urine and pale stools. There was no history of alcohol, drugs or natural products consumption. The patient also denied recent travels. On admission, she was hemodynamically stable and with no fever, abdominal pain or pruritus. Physical examination was positive only for icteric sclera. No other relevant findings including hepatosplenomegaly, ascites or hepatic encephalopathy were found. Also, there were no lymphadenopathies or rash. Blood tests revealed leukopenia of 3.1 × 10^9^ (4.0-11.0 × 10^9^) with normal eosinophil value and thrombocytopenia of 82.000 (150.000-450.000). Immunoglobulin IgE level was normal. Liver tests demonstrated a cytocholestase pattern, with elevated aminotransferases: AST 3170 IU/L (<34 IU/L), ALT 908 IU/L (<49 IU/L), GGT 1132 IU/L (<38 IU/L), alkaline phosphatase (AP) 109 IU/L (<104 IU/L), TB 5.1 mg/dl (<1.0 mg/dl) and DB 4.46 mg/dl (<0.2 mg/dl), as well as prolonged prothrombin time: 20s. (<11,6 s.) and APTT 53 s. (< 31 s.) Abdominal ultrasound showed a homogeneous liver, with regular borders. Only steatosis was evident and no nodular or mass lesions were observed.

**Fig. 1:**
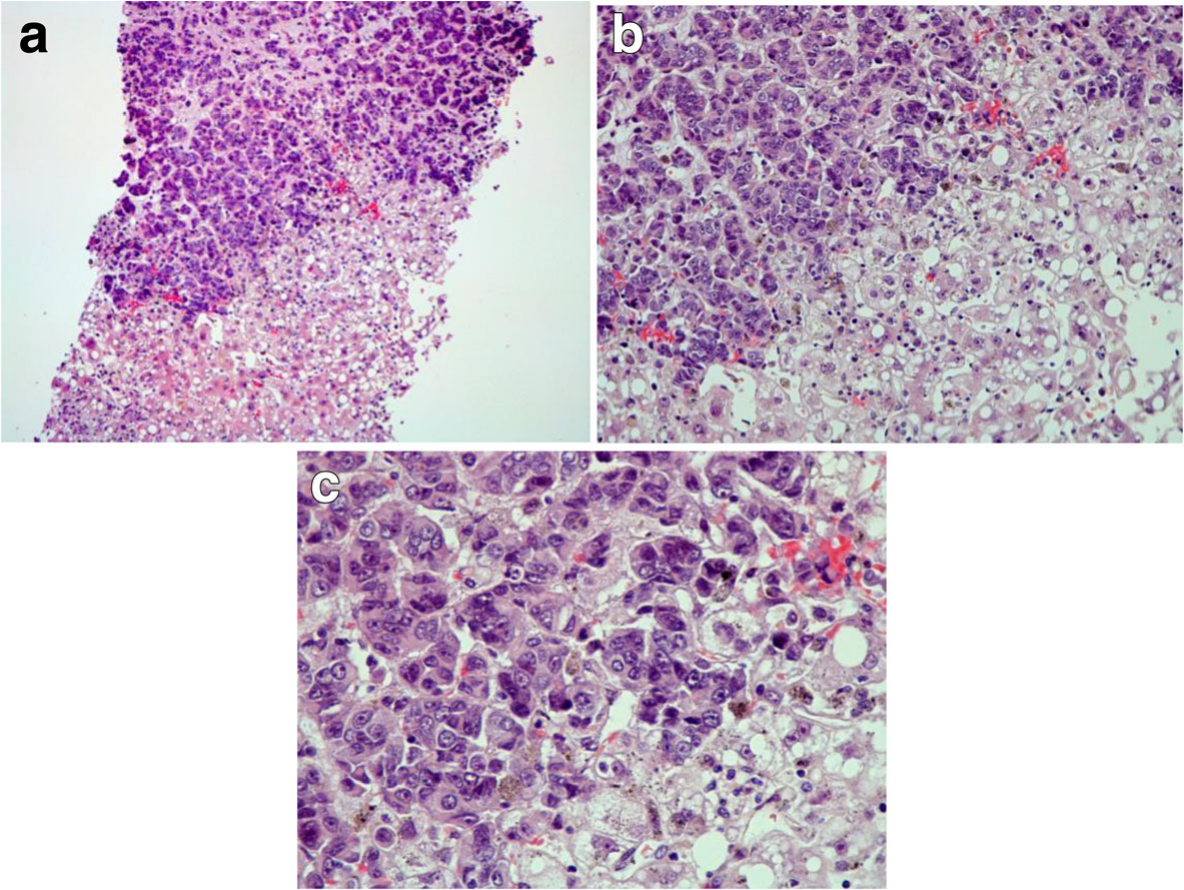
Liver histological findings. **a** Diffuse infiltration by a poorly differentiated adenocarcinoma. **b** The carcinoma cells were arranged singly, in small clusters. **c** Evidence of nucleoli and multiple mitosis suggesting a high proliferation index

**Fig. 2:**
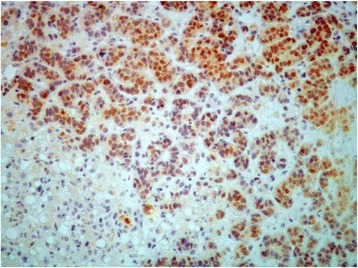
Positive immunohistochemical stains for estrogen receptor and negative for progesterone and Herb-2 receptors

An extensive workup diagnosis including chest CT, viral serologies (HAV, HBV, HCV, CMV), and autoimmune and metabolic studies failed to show an etiology for the disease. A presumptive diagnosis of liver toxicity to Fulvestrant was assumed. Although the drug was immediately withdrawn, clinical worsening occurred with the development of hepatic encephalopathy and rapid progression of acute liver failure (factor V 35%, AST 5530 IU/L, ALT 1810 IU/L, TB 8.6 mg/dl, APTT 55 s. and PT 23.4 s.) associated with bleeding diathesis. Because the patient had an active tumor recurrence which is a contraindication for liver transplant and chemotherapy wasn’t possible because of severe abnormal liver function, a transjugular liver biopsy wasn’t considered as it wouldn’t change the management of the patient. There was no response to medical support treatment and the patient died. Post-mortem examination revealed a wide hepatic infiltration by neoplastic tissue with morphologic characteristics compatible with adenocarcinoma (Fig. 1a). The carcinoma cells were arranged singly in small clusters (Fig. 1b) with a high proliferation index (Fig. 1c). Immunohistochemical stains were positive for estrogens receptor but negative for progesterone and Herb2 receptors (Fig. 2), consistent with the diagnosis of primary breast carcinoma. Unfortunately, E-cadherin and CD44 stains weren’t tested.

## Discussion and conclusions

Although Fulvestrant is a steroidal antiestrogen and it can be associated with liver enzyme elevations, there is no cases of ALF described in literature. In case described liver biopsy excluded this hypothesis. Although liver metastasizing is a common phenomenon, ALF from the spread of the tumor is rare and can be a diagnostic challenge. Clinical, laboratory and radiologic findings are often nonspecific and inconclusive, as it were the case with our patient [1]. According to the largest review of ALF, only 32 cases induced by diffuse metastatic breast cancer have been reported between 1950 and 2014 [6]. The average age described is 47.9 ± 9.9 years such as in the case reported [6]. Most of reported cases have occurred in patients with a previous history of known and treated breast cancer. Ductal carcinoma, like in the present case, is the most frequent one [6]. Our patient had non-specific prodromal symptoms, such malaise and nausea 2 weeks prior to the onset of liver failure, as it has been described [4]. Typically, liver metastases are asymptomatic with a focal/target pattern of parenchymal infiltration on ultrasonography and contrast-CT [2]. However, in ALF, as in case reported, imaging may be not specific, without any focal abnormality of the liver parenchyma. In contrast, in such cases tumor cells invade diffusely liver sinusoids, without involving the parenchyma. Thus, liver surface is smooth, with a preserved shape and with no nodularity, despite an important degree of tumor infiltration. Hence, due to diffuse nature of spread typical findings are not seen on radiologic imaging [2] and the majority of the cases of ALF induced by metastatic infiltration are diagnosed after death [2, 7]. In a recent review of literature only 25% of cases were diagnosed premortem [6]. However, a significant trend for increased *premortem* diagnosis has been noticed [6]. Several mechanisms have been proposed to explain the molecular mechanism of massive liver infiltration and hepatocellular injury that occur in such cases. First, loss of cellular adhesion molecules expression as E-cadherin and CD44 can be involved in this process. The first one, being a cell-to-cell and cell-to-extracellular-matrix adhesion protein, when absent allows cell detachment from the primary tumor and single-cell infiltration. On the other hand, CD44 is responsible for endothelium adhesion and its absence may reflect an inability of the metastatic cells to invade beyond the sinusoidal endothelium preventing the formation of a mass in the parenchyma (intrasinusoidal pattern) [5, 8, 9]. Also, invasion of hepatic vessels by tumor cells may result in ischemia with neoplastic cells exerting a pressure effect on hepatocytes competing for nutrients and oxygen, finally leading to liver cell necrosis [2]. Hepatic metastases, including from breast cancer, can also mimic cirrhosis [7, 9]. This “pseudocirrhosis” or carcinomatous cirrhosis is the result of a desmoplastic response of the liver. Due to the infiltrating tumor and the associated inflammation, stellate cells are activated and produce collagen, causing an extensive fibrosis that results in the atrophy of hepatocytes [5–9].

Most cases of ALF from neoplastic invasion have an extremely poor prognosis, with death occurring from 3 days to 6 months after presentation [2]. The mortality rate of breast carcinoma with diffuse hepatic infiltration is 3 days to 2 months after presentation in 90% of the cases. [2]. *Premortem* diagnosis is important because it affects therapy. Hepatic transplantation is contraindicated for this etiology and chemotherapy is limited by severally abnormal liver function and multiorganic failure [5]. Nonetheless, there are a few clinical cases of patients with metastatic disease unrecognized prior to transplantation that underwent liver transplantation and adjuvant chemotherapy with success [3]. There are also two cases of short-term reversal of ALF from metastatic breast cancer after chemotherapy with longer survival (9 months) [5, 10].

In conclusion, we report a case of a rare cause of ALF induced by metastatic breast cancer. Although rare, physicians must have a high index of suspicion to consider this etiology when approaching a case of ALF, as diffuse intrasinusoidal hepatic metastases of breast cancer can infiltrate the liver with imaging studies remaining normal. There may be a role for liver transplantation in strict selected patients.

## Abbreviations

ALF: Acute liver failure
CMV: Cytomegalovirus
HAV: Hepatitis A virus
HBV: Hepatitis B virus
HCV: Hepatitis C virus
